# Walnut Kernel Oil and Defatted Extracts Enhance Mesenchymal Stem Cell Stemness and Delay Senescence

**DOI:** 10.3390/molecules28176281

**Published:** 2023-08-28

**Authors:** Marwa A. Elsied, Zeina W. Sharawi, Hadba Al-Amrah, Rabab A. Hegazy, Amro E. Mohamed, Rasha M. Saleh, Sanad S. El-kholy, Foad A. Farrag, Masoud H. Fayed, Mohammed A. El-Magd

**Affiliations:** 1 Anatomy and Embryology Department, Faculty of Veterinary Medicine, Kafrelsheikh University, Kafr El-Sheikh 33516, Egypt; 2 Biological Sciences Department, Faculty of Sciences, King AbdulAziz University, Jeddah 21589, Saudi Arabia; zsharawi@kau.edu.sa (Z.W.S.); hggaber@kau.edu.sa (H.A.-A.); 3 Department of Biology, Al-Darb University College, Jazan University, Jazan 45142, Saudi Arabia; rhegazy@jazanu.edu.sa; 4 Biochemistry Division, Chemistry Department, Faculty of Science, Tanta University, Tanta 31527, Egypt; amr.elsherbini@science.tanta.edu.eg; 5 Department of Physiology, Faculty of Veterinary Medicine, Mansura University, Mansura 35516, Egypt; rasha_physiology@mans.edu.eg; 6 Department of Physiology, Faculty of Medicine, Kafrelsheikh University, Kafr El-Shaikh 33516, Egypt; sanad_elkholy2014@med.kfs.edu.eg

**Keywords:** walnut kernel extracts, mesenchymal stem cells, stemness, senescence, antioxidant

## Abstract

Decreased stemness and increased cellular senescence impair the ability of mesenchymal stem cells (MSCs) to renew themselves, change into different cell types, and contribute to regenerative medicine. There is an urgent need to discover new compounds that can boost MSCs’ stemness and delay senescence. Therefore, this study aimed to investigate the impact of walnut kernel oil (WKO) and defatted (WKD) extracts on bone marrow (BM)-MSC stemness and senescence. Premature senescence and inflammation were induced in BM-MSCs using H_2_O_2_ and LPS, respectively. Phytochemical constituents of WKO and WKD extracts were detected by HPLC. The stemness (proliferation and migration), senescence-related markers (p53, p21, SIRT1, and AMPK), oxidative stress/antioxidant markers, inflammatory cytokines, and cell cycle of BM-MSCs were measured by MTT assay, qPCR, ELISA, and flow cytometry. WKO and WKD extracts improved rat BM-MSC stemness, as evidenced by (1) increased cell viability, (2) decreased apoptosis (low levels of *Bax* and caspase3 and high levels of *Bcl2*), (3) upregulated *MMP9* and downregulated *TIMP1* expression, and (4) cell cycle arrest in the G0/G1 phase and declined cell number in the S and G2/M phases. Additionally, WKO and WKD extracts reduced rat BM-MSC senescence, as indicated by (1) decreased *p53* and *p21* expression, (2) upregulated expression and levels of SIRT1 and AMPK, (3) reduced levels of ROS and improved antioxidant activity (higher activity of CAT, SOD, and GPx and upregulated expression of *NrF2* and *HO-1*), and (4) declined levels of TNFα, IL1β, and NF-κB. When compared to the WKO extract, the WKD extract had a greater impact on the induction of stemness and reduction of senescence of BM-MSCs due to its stronger antioxidant activity, which could be attributed to its higher levels of flavonoids and phenolic compounds, as detected by HPLC analysis. WKO and WKD extracts enhance rat BM-MSC stemness and protect them from senescence, suggesting their potential use as enhancers to increase MSCs’ therapeutic efficacy.

## 1. Introduction

Mesenchymal stem cells (MSCs) can self-renew and differentiate and are promising for use in regenerative medicine because of their excellent immunoregulatory, lineage-specific differentiation, and regenerative features [1,2]. However, before their full therapeutic promise can be realized, much more research into their biology and safety is required [3]. A major challenge in MSC-based therapy is the loss of stemness (proliferation and homing capability) due to cellular senescence. The latter is caused by many stresses, including telomere shortening, DNA damage, and ROS [4]. Based on these stressors, senescence was divided into two tyspes: replicative senescence produced by damaged telomeres, and premature senescence triggered by DNA damage and ROS [5]. Premature senescence is characterized by high levels of p53 and p21 [6]. This prompted scientists to spend the last decade searching for novel compounds to enhance MSCs’ stemness and reduce senescence. Preconditional treatment of MSCs by enhancers, such as pharmacological substances, growth factors, hormones, and natural bioactive compounds, boosts MSCs’ therapeutic potentials [7]. Among these enhancers, the antioxidant polyphenol resveratrol, found in peanuts, walnuts, and the peel of red grapes, has been shown to increase MSCs’ stemness and therapeutic effectiveness and reduce senescence [5,8] through upregulation of sirtuin 1 (SIRT1) and downregulation of senescence- and inflammation-related markers (p53, p21, NFα, IL1β, and NF-κB) [5,9,10]. Resveratrol-treated MSCs possessed high antioxidant properties through the SIRT1/Akt pathway in cardiomyopathic rats [11]. SIRT1 is a type of class III HDAC that regulates epigenesis and cellular senescence. It also can inhibit MSC senescence via suppression of the p53/p21 pathway and activation of Akt [12]. Activation of the AMPK pathway also improved MSC stemness. Metformin induced differentiation of dental pulp-derived MSCs into bone cells via activation of the AMPK pathway [13].

Finding new compounds from natural products for disease remedies is a common practice in medicine, as most discovered drugs are originated from medicinal plants. The walnut (*Juglans regia* L.) is distributed all over the world due to its commercially valuable kernels/seeds [14]. The walnut fruit consists of an external green husk (epicarp), a nut that contains a light brown shell (endocarp), and the edible seed (kernel), which is covered by a septum (pellicle) [15]. Walnut green husks have antioxidant characteristics and are utilized to cure pain and inflammation [16]. The walnut kernel (WK) has nutritional and health-promoting values due to its high content of phytochemicals with potent antioxidant activity such as unsaturated fatty acids (linoleic, oleic, and linolenic), phenolic compounds, tocopherols, phospholipids, sphingolipids, sterols, hydrocarbons, and volatile compounds [17,18,19,20]. Regular and sufficient WK consumption reduces the risk of cancer, heart diseases, and other chronic degenerative disorders [21]. Antioxidant phytochemicals included in WK extracts protect from the destructive effects of ultraviolet (UV) radiation and microbial infection [22,23], are used as natural antioxidants to preserve cooked sausages [24], and prevent hemolysis with no cytotoxic effects on blood platelets [20,23]. Moreover, WK extracts and their derivatives have strong cancer-fighting properties against many different types of human cancer cells such as HepG2, HCT-116, A549, A498, CaCo2, MCF-7, BEL-7402, HeLa, COLO205, BGC-823, and SKOV3 [20,25,26,27]. Rusu, et al. [28] also reported the anticancer potential for WK septal extract against lung and breast cancer cells, but they did not find cytotoxic effects on normal human gingival fibroblast cells.

So far, only one study has evaluated the effect of extracts prepared from the internal septum of the WK on rat BM-MSCs and found a cytotoxic effect [29]. Another report denoted a cytotoxic effect of walnut lipid extracts on colorectal cancer stem cells (CSCs) [19,30]. The scientific literature lacks evidence on how WK extracts influence BM-MSC stemness and senescence. Therefore, this study was conducted to investigate this impact.

## 2. Results

### 2.1. Flavonoids and Phenolic Compounds of WKO and WKD Extracts as Detected by HPLC

**Figure 1:**
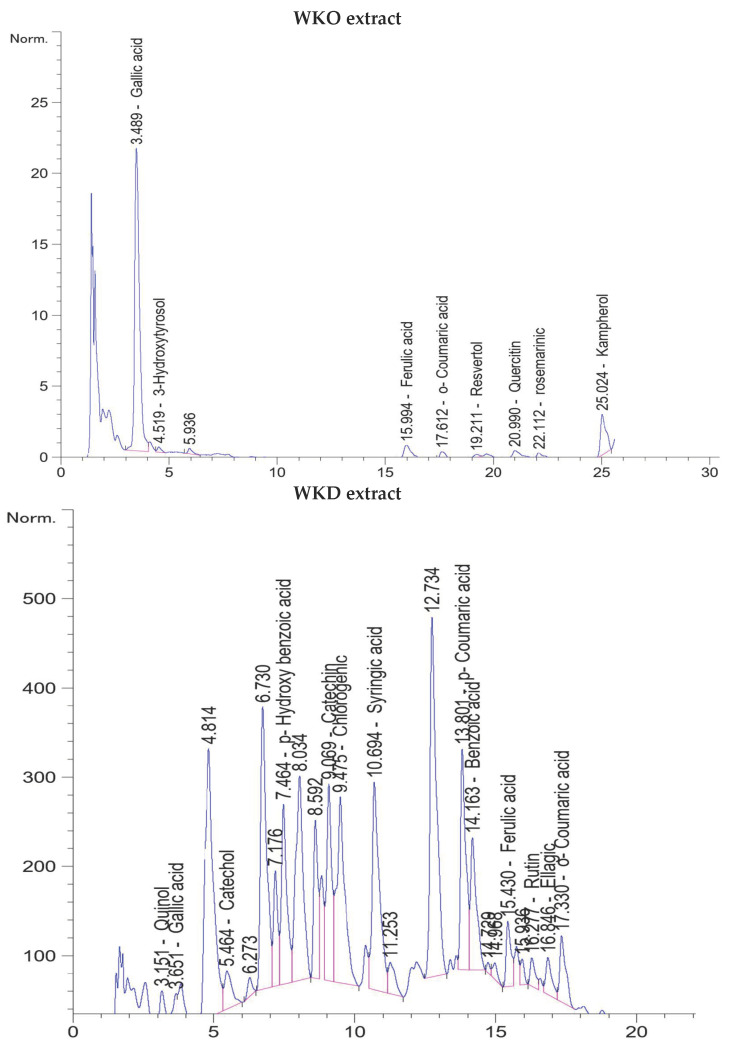
HPLC chromatograms of WKO and WKD extracts showed the presence of various phenolic and flavonoid compounds.

HPLC analysis of the WKO extract revealed the presence of various flavonoids and phenolic compounds. Based on retention time from lowest to highest, these compounds were gallic acid, 3-hydroxytyrosol, ferulic acid, o-coumaric acid, resveratrol, quercetin, rosmarinic acid, and kaempferol. Based on the concentration, the highest three compounds were gallic acid (21.81 µg/g at 3.489 min), resveratrol (9.4 µg/g at 19.2 min), and 3-hydroxytyrosol (3.49 µg/g at 4.5 min) (Table 1, Figure 1). On the other hand, the WKD extract’s HPLC chromatograms showed the presence of various flavonoids and phenolic compounds, including quinol, gallic acid, catechol, p-hydroxybenzoic acid (a phenolic derivative of benzoic acid), catechin, chlorogenic acid, syringic acid, p-coumaric acid, benzoic acid, ferulic acid, rutin, and ellagic and o-coumaric acid. Benzoic acid was the most abundant phenolic compound (280.00 µg/g at 14.16 min), followed by p-hydroxybenzoic acid (73.74 µg/g at 7.46 min), gallic acid (58.50 µg/g at 3.65 min), quinol (52.26 µg/g at 3.15 min), and chlorogenic acid (38.47 µg/g at 9.47 min), whereas catechol (21.93 µg/g at 5.46 min) was the most abundant flavonoid, followed by rutin (19.02 µg/g at 16.27 min) (Table 1, Figure 1). The phenolic and flavonoid content of WKD extract was much greater than that of WKO extract (618.37 µg/g vs. 43.93 µg/g, respectively).

### 2.2. Isolation and Identification of Stem Cells

**Figure 2:**
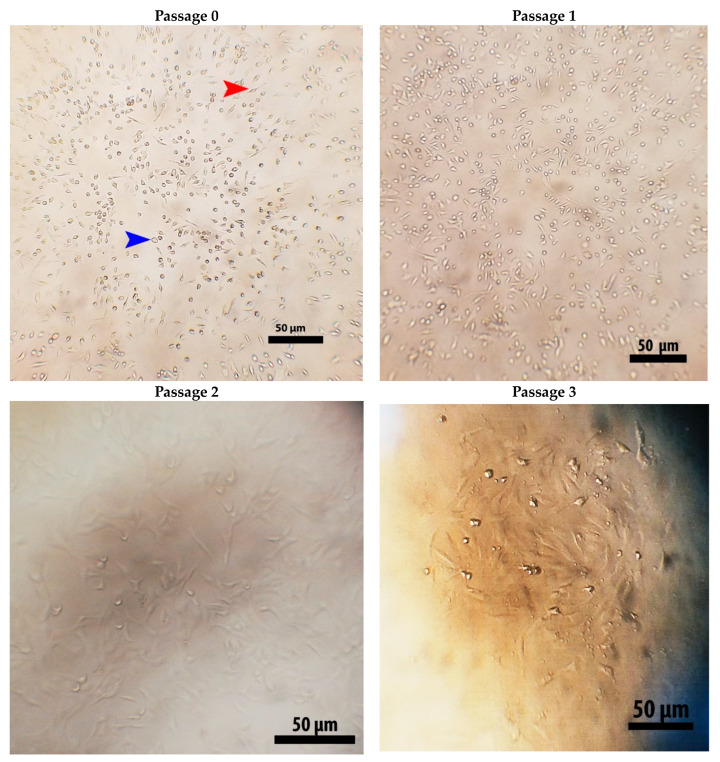
Cultured rat BM-MSCs on the first day of isolation (passage 0) and the successive three passages with near confluences of 50% (passage 0), 70% (passage 1), 80% (passage 2), and 85% (passage 3) showing distinctive fusiform adherent (red arrowhead) and non-adherent round cells (blue arrowhead). In passage 3, MSCs had a typical fusiform (fibroblast-like) shape and were used in further experiments. Scale bar = 50 μm.

**Figure 3:**
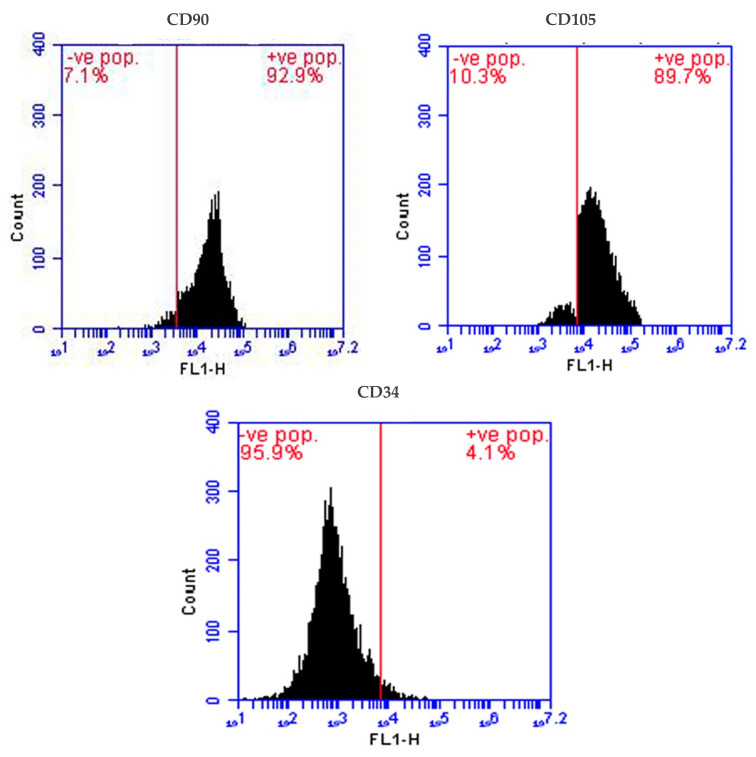
Flow cytometry validation for rat BM-MSC isolation as revealed by the presence of higher MSC-specific markers CD90+ (93%) and CD105+ (89%), and lower hemopoietic stem cell marker CD34 (4%).

MSCs were isolated from young male rat bone marrow and their identity was confirmed by inverted microscope examination (fusiform cells adhered firmly to the bottoms of the tissue culture flasks) (Figure 2) and by flow cytometry, which confirmed the presence of a high number of cells expressing MSC-specific surface markers CD90 (93% positive) and CD105 (89% positive), and a very low number of cells expressing hemopoietic stem cell marker CD34 (4%) (Figure 3).

### 2.3. WKO and WKD Extracts Improved BM-MSC Stemness 

#### 2.3.1. WKO and WKD Extracts Enhanced BM-MSC Proliferation

**Figure 4:**
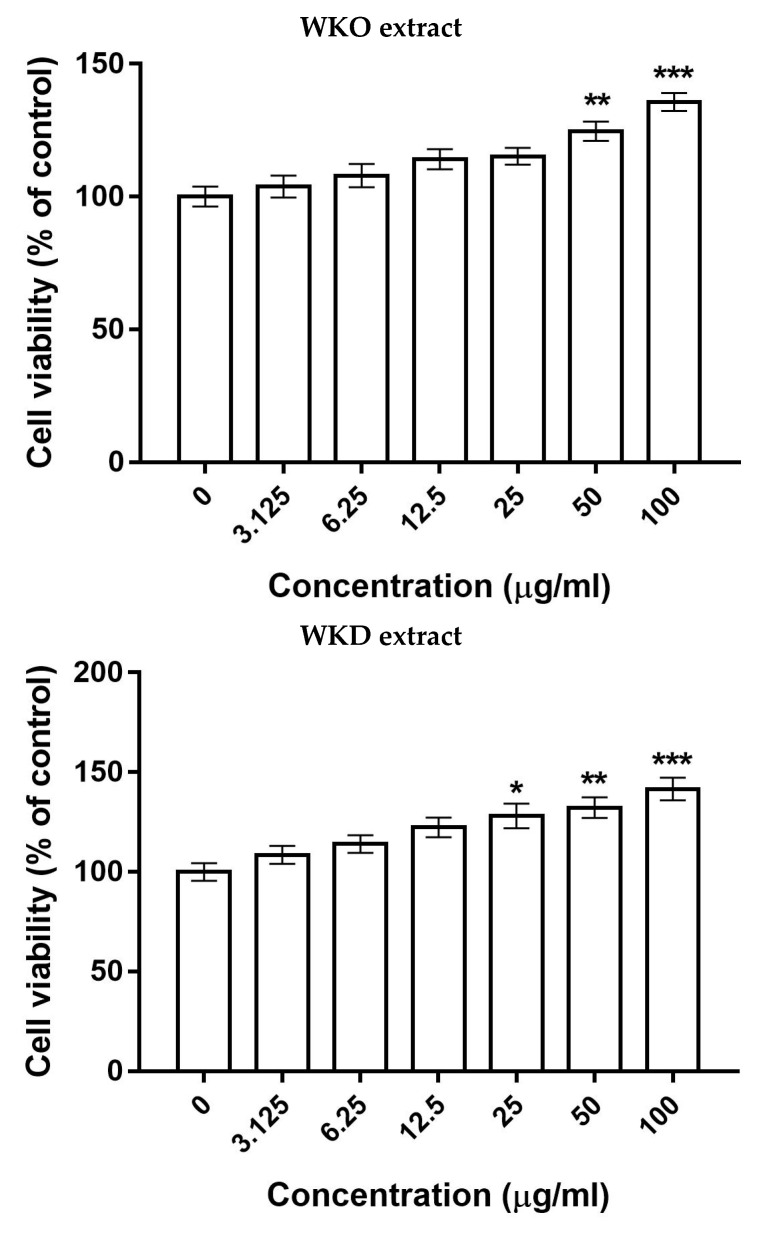
WKO and WKD extracts induced a significant increase in MSC viability at concentrations of 50 and 25 µg/mL, respectively, as detected by MTT assay. Data are expressed as mean ± SEM, *n* = 3. All doses were compared to the untreated cells (0), with significance set at * *p* ≤ 0.05, ** *p* ≤ 0.01, and *** *p* ≤ 0.001.

The proliferation of rat BM-MSCs was detected 24 h after incubation with several doses of WKO or WKD extracts using the MTT assay. The WKO extract significantly (*p* ≤ 0.05) induced rat BM-MSC proliferation at a concentration starting from 50 µg/mL, whereas the WKD extract significantly (*p* ≤ 0.05) triggered the proliferation at a lower concentration of 25 µg/mL (Figure 4). For this reason, both extracts were used in the further experiments at a dose of 50 µg/mL.

#### 2.3.2. WKO and WKD Extracts Inhibited Apoptosis and Induced Migration of BM-MSCs

**Figure 5:**
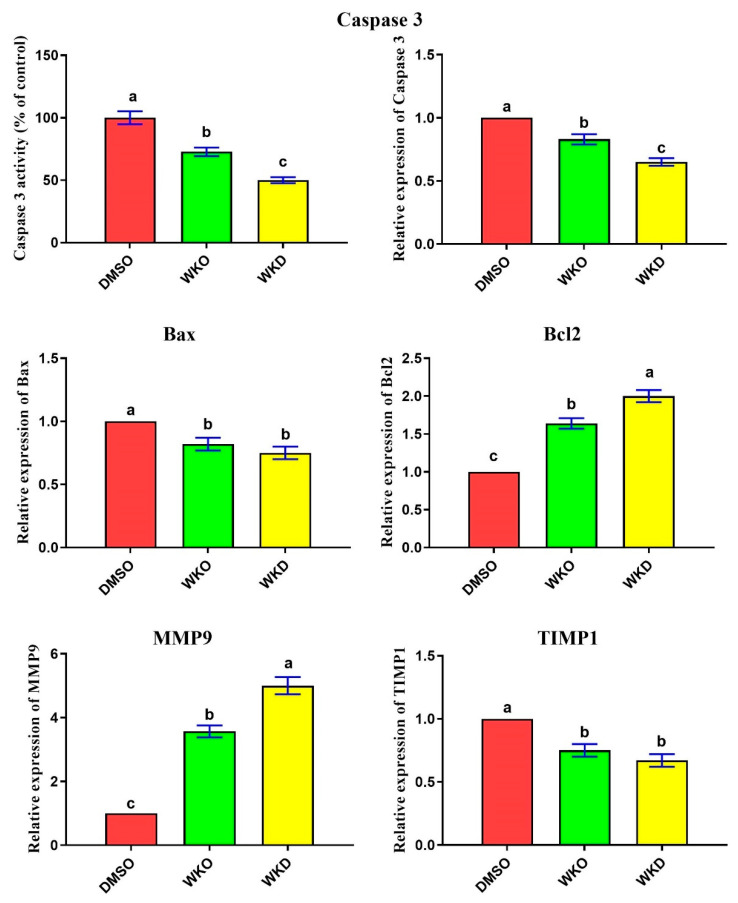
WKO and WKD extracts inhibited apoptosis and induced migration of rat BM-MSCs. Effect of the two extracts on caspase 3 activity/expression and gene expression of *Bax*, *Bcl2*, *MMP9*, and *TIMP1* as determined by ELISA and real-time qPCR. Data are expressed as mean ± SEM, *n* = 3. Letters a–c denote significant differences between the three groups at *p* < 0.05. Each group was compared against every other group. DMSO, vehicle-treated control cells; WKO, walnut kernel oil extract-treated cells; WKD, walnut kernel defatted extract-treated cells.

To check whether the proliferative effect of WKO or WKD extracts could be associated with an anti-apoptotic potential, we first detected their effect on the activity of cleaved caspase 3 (as the end-product of apoptosis) using ELISA. We found significantly (*p* ≤ 0.05) lower levels of cleaved caspase 3 in BM-MSCs treated with each extract, with the lowest levels in the WKD group, than in the control (DMSO) group (Figure 5). We then evaluated the effect of the two extracts on apoptosis-related genes (*Bax* and caspase 3) and the anti-apoptotic *Bcl2* gene and found significantly (*p* ≤ 0.05) decreased expression of *Bax* and caspase 3 and significantly increased expression of *Bcl2* in the WKO and WKD groups, with the best effect in the WKD group, relative to the DMSO group (Figure 5). Additionally, WKO or WKD extracts induced rat BM-MSC migration, as revealed by significantly (*p* ≤ 0.05) upregulated expression of the migration-related *MMP9* gene and significantly (*p* ≤ 0.05) downregulated expression of the anti-migration *TIMP1* gene in the WKO and WKD groups, with the best effect in the WKD group, compared to the DMSO group (Figure 5).

#### 2.3.3. WKO and WKD Extracts Affected BM-MSC Cell Cycle

**Figure 6:**
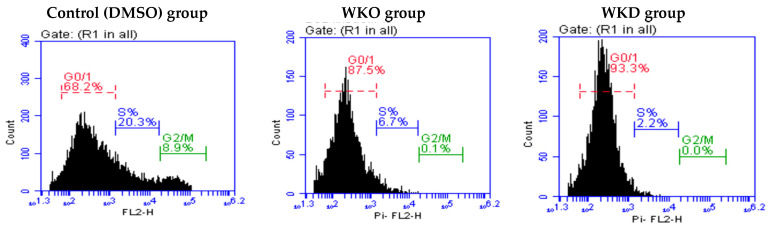
Effect of WKO and WKD extracts on cell cycle phases (G0/G1, S, and G2/M) of MSCs as detected by PI-labeled flow cytometry histograms in three representative samples, one from each group. The number of cells per phase is presented as %. DMSO, vehicle-treated control cells; WKO, walnut kernel oil extract-treated cells; WKD, walnut kernel defatted extract-treated cells.

Flow cytometry with PI labeling was used to examine the effect of WKO and WKD extracts on the cell cycle of rat BM-MSCs, and the results are shown in Table 2 and Figure 6. Cell cycle arrest in the G0/G1 phase was found in MSCs treated with either WKO or WKD extracts, as revealed by the increased number of MSCs in these two groups (88.4 ± 0.9% and 92.2 ± 1.1%, respectively) compared to the control (DMSO) cells (70.7 ± 2.5%). However, the number of cells in the S and G2/M phases was significantly (*p* ≤ 0.05) lower in the WKO and WKD groups than in the DMSO group.

### 2.4. WKO and WKD Extracts Reduced BM-MSC H_2_O_2_-Induced Premature Senescence 

#### 2.4.1. WKO and WKD Extracts Repressed BM-MSC Senescence-Related Markers

**Figure 7:**
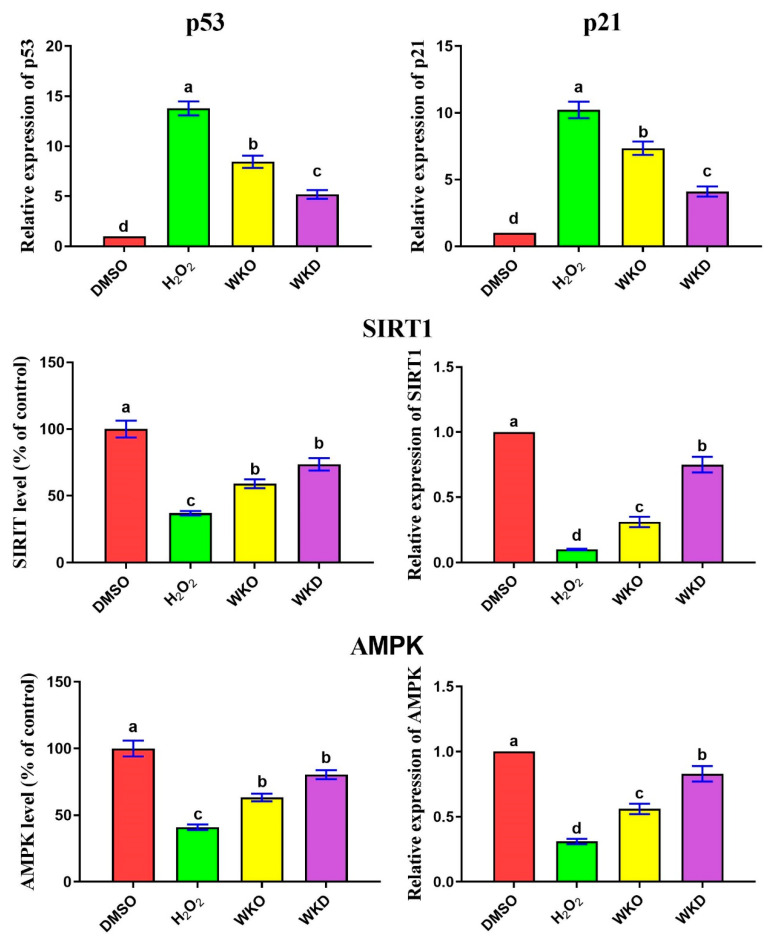
WKO and WKD extracts repressed BM-MSC senescence-related markers. Effect of the two extracts on SIRT1 and AMPK levels/expression and gene expression of *p53* and *p21* as measured by ELISA and qPCR. Data are expressed as mean ± SEM, *n* = 3. Letters a–d indicate significant differences between the three columns at *p* < 0.05. Values (columns) with the same letters are insignificant. All groups were compared to each other. DMSO, vehicle-treated control cells; H_2_O_2_, H_2_O_2_ -treated cells; WKO, walnut kernel oil extract-treated cells; WKD, walnut kernel defatted extract-treated cells.

As mentioned earlier, senescent BM-MSCs have upregulated expression of senescence-related genes (*p53*, *p21*) and downregulated expression of *SIRT1* and *AMPK*. As expected, cells treated with H_2_O_2_ possessed significantly (*p* ≤ 0.05) upregulated expression of *p53* and *p21* and significantly (*p* ≤ 0.05) downregulated expression and levels of SIRT1 and AMPK compared to the control (DMSO) cells (Figure 7). In contrast, BM-MSCs pretreated with WKO or WKD extracts showed significantly (*p* ≤ 0.05) declined *p53* and *p21* expression and significantly increased expression and levels of SIRT1 and AMPK relative to cells treated with H_2_O_2_ alone. However, treatment with WKO or WKD extracts failed to return to *p53*, *p21*, SIRT1, and AMPK levels comparable to the control group.

#### 2.4.2. WKO and WKD Extracts Protected BM-MSCs against H_2_O_2_-Triggered ROS

**Figure 8:**
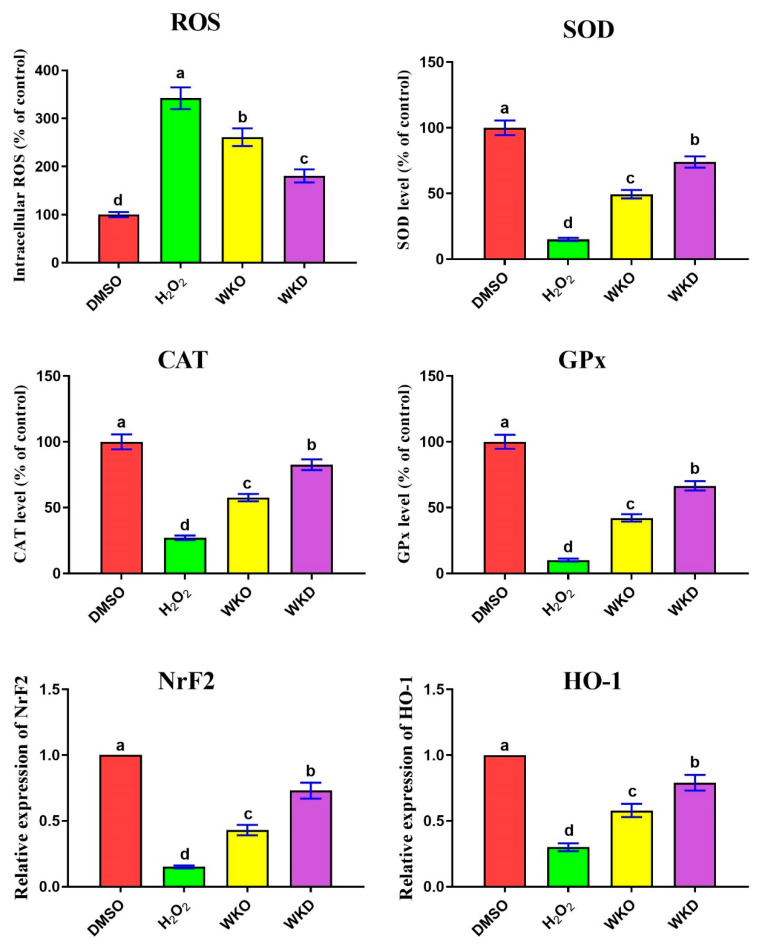
WKO and WKD extracts protected BM-MSCs against H_2_O_2_-triggered ROS. Effects of the two extracts on intracellular ROS, activity of antioxidant enzymes (CAT, SOD, GPx), and expression of *NrF2* and *HO-1* genes were detected in BM-MSCs. Data are presented as mean ± SEM, *n* = 3. Letters a–d indicate significant differences between the four groups at *p* < 0.05. Values (columns) with the same letters are insignificant. All groups were compared to each other. DMSO, vehicle-treated control cells; H_2_O_2_, H_2_O_2_-treated cells; WKO, walnut kernel oil extract-treated cells; WKD, walnut kernel defatted extract-treated cells.

We hypothesized that WKO and WKD extracts may decrease ROS induced by H_2_O_2_ and raise endogenous antioxidant enzyme activity due to the presence of many flavonoids and phenolic compounds in these extracts. To test this hypothesis, we measured intracellular ROS (as a prominent indicator of oxidative stress) and the activity of antioxidant enzymes (CAT, SOD, GPx) in BM-MSCs treated with the ROS inducer H_2_O_2_ and/or each of the WKO and WKD extracts. As shown in Figure 8, cells treated with H_2_O_2_ alone showed significantly (*p* ≤ 0.05) higher intracellular ROS levels and significantly (*p* ≤ 0.05) lower activity of antioxidant enzymes than the control (DMSO) cells. However, the levels of CAT, SOD, and GPx in cells pre-treated with each extract were significantly (*p* ≤ 0.05) greater, whereas intracellular ROS levels were significantly (*p* ≤ 0.05) reduced compared to cells treated with H_2_O_2_ alone. The antioxidant potential of the two extracts was further verified at mRNA levels as detected by qPCR, which showed significantly (*p* ≤ 0.05) abundant expression of the antioxidant *NrF2* gene and its downstream target *HO-1* in BM-MSC cells pre-treated with each extract, with the lowest levels in WKD cells, relative to those treated with H_2_O_2_ alone (Figure 8).

#### 2.4.3. WKO and WKD Extracts Protected BM-MSCs against LPS-Induced Inflammation

**Figure 9:**
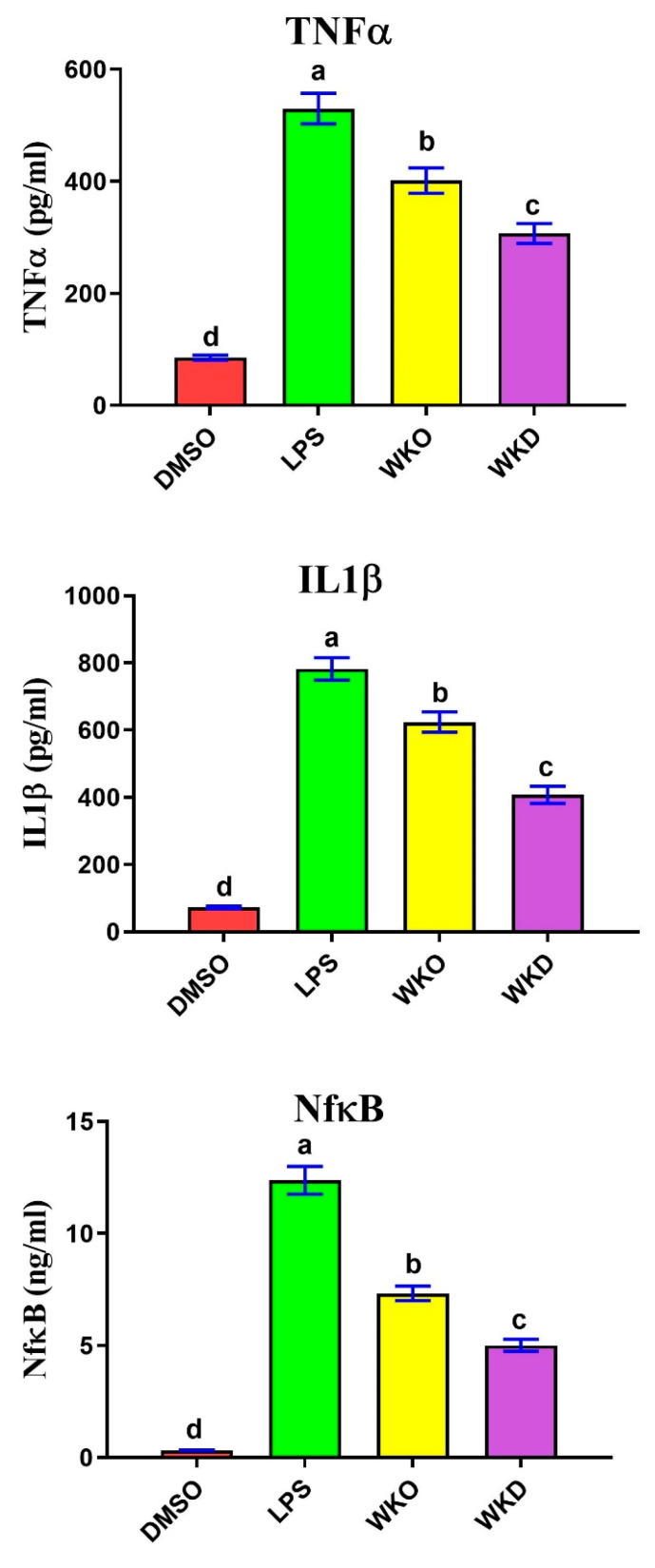
WKO and WKD extracts protect rat BM-MSCs against inflammation induced by LPS. Effect of the two extracts on the levels of pro-inflammatory cytokines TNFα, IL1β, and NF-kB as measured by ELISA. Values are presented as mean ± SEM, *n* = 3. Letters a–d denote significant differences between the four groups at *p* < 0.05. Each group was compared against every other group. DMSO, vehicle-treated control cells; LPS, lipopolysaccharides-treated group; WKO, walnut kernel oil extract-treated cells; WKD, walnut kernel defatted extract-treated cells.

To detect the protective potential of WKO and WKD extracts on lipopolysaccharides (LPS) -induced inflammation, ELISA was used to measure the change in the concentrations of pro-inflammatory cytokines TNFα, IL1β, and NF-kB, and the results are shown in Figure 9. Expectedly, BM-MSCs treated with LPS alone exhibited significantly (*p* ≤ 0.05) higher levels of the three cytokines than the control (DMSO) cells. In contrast, cells pretreated with WKO or WKD extracts showed significantly (*p* ≤ 0.05) decreased levels of TNFα, IL1β, and NF-kB, with the lowest levels in WKD-treated cells, relative to LPS-treated cells. However, the declined levels of these cytokines remained significantly (*p* ≤ 0.05) higher than those of the control cells.

## 3. Discussion

Walnut seed/kernel has nutritional and health-promoting values due to its high content of phytochemicals with potent antioxidant effects [17,18]. Although MSCs show great promise for tissue regeneration and repair, there is still much to learn about their biology, manipulation, and safety [3]. To improve MSCs’ regenerative potential, new compounds are needed to enhance their stemness and reduce their senescence. Herein, we examined the effect of WKO and WKD extracts on rat BM-MSC stemness and senescence. To the best of our knowledge, this is the first study to report that WKO and WKD extracts could enhance MSC stemness and reduce premature senescence. 

In the present study, we isolated MSCs from the bone marrow of the femur of 4-month-old male rats and confirmed their successful isolation using an inverted microscope, which confirmed MSC identity based on their morphology and firm adhesion to the bottoms of the tissue culture flasks [29,31,32]. Due to the substantial reduction in adhering hematopoietic stem cell lineages after 72 h of initiation culture [32], we employed MSCs obtained after passage 3. We then confirmed the high number of isolated MSCs by detection of MSC-specific surface markers CD90 (93%) and CD105 (89%) and the lack of CD34-negative markers (4%) by flow cytometry. Similarly, Alzahrani et al. [31] and Aghapour SK and Sisakhtnezhad [29] found 99.15 and 97.33% CD105 and 91.50 and 71.33% CD90 but just 1.72 and 1.46% CD34, respectively, for isolated rat BM-MSCs.

MSCs stemness refers to MSCs’ ability to proliferate and migrate (homing). To study the effects of WKO and WKD extracts on rat BM-MSC viability, we performed the MTT assay and found that both extracts induced MSC proliferation. These findings imply that these extracts had no cytotoxic effect on MSCs but rather triggered MSC proliferation. To check whether this proliferative effect is related to anti-apoptotic potential, we used qPCR and ELISA and found a significant downregulation of the apoptotic (*Bax* and caspase3) genes and reduced cleaved caspase3 activity and a significant upregulation of the anti-apoptotic *Bcl2* gene in MSCs treated with WKO or WKD extracts. These results suggest that the proliferative effect of WKO or WKD extracts on MSCs could be associated with their anti-apoptotic potential on these cells. To the best of our knowledge, this is the first report to denote that WKO or WKD extracts have proliferative and anti-apoptotic effects on MSCs. Unlike that of the walnut kernel, extracts prepared from other parts of the walnut showed different effects. Indeed, aqueous decoction of the internal septum of the walnut kernel (DISWK) showed a cytotoxic effect on rat BM-MSCs with a half-maximal inhibitory concentration (IC_50_) value of 50 and 100 µg/mL after 24 and 48 h incubation, respectively, in a dose- and time-dependent manner [29]. Moreover, another study reported a potent cytotoxic effect of walnut lipid extracts (WLEs) on colorectal cancer stem cells (CSCs) [19,30]. Aside from MSCs and CSCs, Rusu et al. [28] also found that exposure to walnut septal extract (WSE) caused a dose-dependent reduction in lung and breast cancer viability, but it did not affect normal human gingival fibroblast cells. Walnut is rich in jug lone, a phenolic compound that possesses potent inhibitory potential against intestinal cancer in rats [16,33]. Taken together, different walnut parts could have different effects on a large variety of normal cells, MSCs, and cancer cells.

The promotion of MSC migration is another potential mechanism by which WKO and WKD extracts boost their stemness. Migrating MSCs to damaged areas where they can do their job is essential. This homing is regulated by members of MMPs and their inhibitor TIMPs. Stemness was enhanced in MSCs by expressing high quantities of MMP9 mRNA and low levels of TIMP1 mRNA [34,35,36]. In agreement, we also found upregulated expression of the migration-related *MMP9* gene and downregulated expression of the ant-migratory *TIMP1* gene in BM-MSCs treated with WKO or WKD extracts.

Flow cytometry with PI labeling was used to examine the effect of WKO and WKD extracts on the cell cycle of rat BM-MScs, and the results revealed a cell cycle arrest in the G0/G1 phase in MSCs treated with each extract, as indicated by the high number of cells arrested in this phase. Interestingly, we also found a significant decrease in cell number in the S and G2/M phases following treatment with each extract. In agreement, Aghapour SK and Sisakhtnezhad [29] also reported a significant decrease in rat BM-MSC number in the S and G2/M phases after treatment with DISWK for 48 h. Decreased number of cells in the S and G2/M phases after treatment with WKO, WKD, or DISWK extracts could indicate that a large number of MSCs exit the cell cycle and start to differentiate. Indeed, Aghapour SK and Sisakhtnezhad [29] reported that treatment with DISWK extracts increased the rat BM-MSCs’ capacity to differentiate into islet-like pancreatic clusters.

It is well known that as MSCs become senescent, their *SIRT1* and *AMPK* expression levels decrease [37]. In line with this, we found similar lower levels and expression of SIRT1 and AMPK in senescent MSCs. However, following the treatment with WKO and WKD extracts, the expression was restored to a level comparable to control (DMSO-treated) MSCs. Additionally, WKO and WKD extracts downregulated *p53* and *p21* in senescent BM-MSCs induced by H_2_O_2_. In agreement, previous studies also reported the anti-aging impact of SIRT1 on MSCs through inhibition of the p53/p21 pathway [10,12]. *SIRT1* also promotes self-renewal and multipotency of MSCs through the upregulation of *SOX2* and its target genes [38]. SIRT1 can induce BM-MSC differentiation into bone cells through the activation of β-catenin and its downstream target genes that are needed for MSC differentiation [39]. SIRT1 deficiency in MSCs hindered their differentiation into bone cells, whereas SIRT1 over-expression lowered bone loss due to aging in male mice [40,41]. Moreover, AMPK activation was also shown to increase MSC stemness. Metformin activated the AMPK pathway, causing MSCs generated from tooth pulp to differentiate into bone cells [13]. In summary, WKO and WKD extracts may enhance the stemness of MSCs and prevent senescence by upregulating SIRT1 and AMPK and downregulating *p53* and *p21*. However, additional experiments involving the gain and loss of function on SIRT1 and AMPK should be performed to confirm this hypothesis.

Overproduction of ROS causes oxidative stress damage to DNA, proteins, and lipids, ultimately leading to premature cellular senescence [5]. We found that treatment with WKO or WKD extracts reduced levels of intracellular ROS and improved the antioxidant status (higher activity of CAT, SOD, GPx, and upregulated expression of *NrF2* and *HO-1*). Therefore, WKO and WKD extracts may be able to postpone the senescence of MSCs by lowering ROS and increasing antioxidant activity. Consistent with our results, WK and walnut septum extracts induced a potent antioxidant effect on both the D-galactose-induced aging paradigm and naturally aged rats [42]. On the other hand, increased levels of pro-inflammatory cytokines can also trigger cellular senescence. Herein, we documented that treatment of MSCs with WKO or WKD extracts resulted in a reduction of TNFα, IL1β, and NF-κB levels. Similarly, natural products have the potential to reduce MSC senescence via inhibiting pro-inflammatory cytokines [5,8,10,43,44]. Additionally. during BM-MSC differentiation, SIRT1 expression increases through the inhibition of NF-kB [45].

The phytochemicals in WK have significant antioxidant activity, which is largely responsible for its nutritional and health-promoting properties [17,18,19,20]. In accordance, the results of HPLC showed several flavonoids and phenolic compounds in the two extracts, with abundant amounts of gallic acid, resveratrol, and 3-hydroxytyrosol in the WKO extract, and benzoic acid, p-hydroxybenzoic acid, chlorogenic acid, catechol, catechin, and rutin in the WKD extract. Previous studies showed a positive effect of these compounds on MSC differentiation into different cell lineages. Benzoic acid has been shown to induce the differentiation of neural stem cells [46], chlorogenic acid can stimulate osteogenesis in human adipose tissue-derived MSCs (AD-MSCs) [46,47], hydroxyl benzoic acid and catechol can induce adipogenesis of AD-MSCs [48,49], and catechin stimulates differentiation of BM-MSCs into adipocytes [50,51] and AD-MSCs into osteocytes [52]. Resveratrol, a compound present in both WKO and WKD extract, has been shown to increase MSC stemness and decrease senescence [5,8] by increasing SIRT1 and decreasing senescence-related genes (*p53*, *p21*) and inflammatory cytokines NFα, IL1β, and NF-κB [5,9,10]. Resveratrol-treated MSCs served as antioxidants through the SIRT1/Akt pathway [11] and restored MSC differentiation into bone cells by blocking the inflammation triggered by TNFα [10]. It is likely that the flavonoids and phenolic chemicals found in WKO and WKD extracts are responsible for their favorable impact on MSCs (improving stemness and delaying senescence). Comparing the therapeutic potential of the two extracts, cells treated with WKD elicited distinguished improvements in MSC stemness (as indicated by increased cell proliferation and migration and decreased cell apoptosis) and delayed MSC senescence (as evidenced by lower ROS, senescence-related parameters, and higher antioxidant and anti-inflammatory effects) compared to cells treated with WKO. The increased concentrations of flavonoids and phenolic components in WKD extract may explain why it had a better impact. 

The limitations of this study are as follows: (1) the study was only in vitro, which means it does not reflect the complex interactions and environment of the living organism; (2) the study used whole extract rather than individual compound, which means it did not identify the specific active ingredients or the precise mechanisms of walnut extract on MSC stemness and senescence; and (3) the study lacked gain- and loss-of-function experiments to essential pathways such as SIRT1, which is a key regulator of MSC stemness and senescence. Further experiments are required to isolate individual compounds from the walnut extract and evaluate their effects on MSC homing and therapeutic potential in animal models (in vivo study) to determine the actual underlying mechanism of action.

## 4. Materials and Methods

### 4.1. Preparation of Extract

One kilogram of walnuts was bought from a local market in Kafrelsheikh city, Kafrelsheikh governorate, and was authenticated by the Pharmacognosy Department, Faculty of Pharmacy, Kafrelsheikh University. A voucher sample was submitted under number 1-4-2021. Walnut kernel (450 g) was ground using an electric grinder and 3 L of n-hexane was added to obtain walnut kernel defatted (WKD) extract. First, the powder–hexane mixture was stirred thoroughly using a glass rod. Second, the mixture was subjected to an ultrasound homogenizer (A 20 kHz, 500 W, Sonics, Vibra cell, Newtown, CT, USA) programmed to pulse on for 40 s and off for 20 s for a total extraction time of 1 h. Finally, after decanting and vacuum filtering the hexane, it was condensed in vacuo using a rotary evaporator at 45 °C to obtain the hexane–WKD extract. To obtain walnut kernel oil (WKO) extract, the marc (defatted powder) was dried, 3 L of 90% methanol were added, and the mixture was thoroughly mixed before repeating the ultrasound extraction as described earlier to obtain the methanol–WKO extract. The latter was filtered and condensed in vacuo (using a rotary evaporator at 45 °C). The hexane (WKD) and methanol (WKO) extracts were then transferred to a suitable vessel and stored at −20 °C until further use.

### 4.2. Detection of Flavonoids and Phenolic Compounds by HPLC

WKO and WKD extracts were analyzed for flavonoids and phenolic compounds using high-performance liquid chromatography (HPLC, Agilent11260 infinity HPLC series, Agilent Technologies, Santa Clara, CA, USA) equipped with a quaternary pump, a vehicle sampler, a diode array detector, and an akinetex column (5 µm EVO C18, 100 mm × 4.6 mm, Phenomenex, Torrance, CA, USA), operated at 30 °C, as reported before [53]. The separation was achieved using a ternary linear elution gradient with three solvents as follows: HPLC-grade water 0.2% H3PO4 (*v*/*v*, solvent A), methanol (solvent B), and acetonitrile (solvent C). All peaks were seen at 284, 320, and 360 nm wavelengths, and the injection volume was 20 μL. Standards were used to evaluate the peaks.

### 4.3. Isolation and Identification of Stem Cells

Using methods previously reported [31], MSCs were isolated from the bone marrow of the femur and tibia of young adult Wistar albino rats (100 ± 10 g, *n* = 3). Briefly, bone marrow was removed by flushing and washed in sterile phosphate buffer saline (PBS); re-suspended in a complete media including DMEM, 10% fetal bovine serum, and 1% penicillin/streptomycin (all from Lonza Bioproducts, Basel, Switzerland); and centrifuged at 2500 rpm for 7 min. Isolated bone marrow cells were cultivated at 37 °C with 5% CO_2_ in a tissue culture incubator, with the media changed often to remove non-adherent cells. Adherent cells at 80% confluence were detached using trypsin (Trypsin-EDTA 1X, Gibco) for 2 min at room temperature, and the obtained MSCs after passage 3 were used in further experiments.

Using specific stem cell markers, including two positive markers—anti-CD105 (1:100 dilution) and anti-CD90 (1:200 dilution), and one negative marker—anti-CD34 (1:100, a mark for hematopoietic cells), the obtained cells were verified as MSCs by flow cytometry (FACScan, BD Biosciences, One Becton Drive Franklin Lakes, NJ, USA) as directed by the manufacturer.

### 4.4. MTT-Based Cell Proliferation Assay

In a 96-well plate, 10,000 BM-MSCs per well equivalent to 100 μL/well were cultured in a complete media and incubated at 37 °C/5% CO_2_/24 h until 80–90% confluence was reached. Before being added to plates, each extract was first solubilized in dimethylsulfoxide (DMSO) to create 50 mg extract/mL stock solutions. WKO and WKD extracts were separately added at serial dilutions (0, 12.5, 25, 50, 100, and 200 μg/mL) and BM-MSCs were incubated for 24 h. The final DMSO concentration was kept below 1% throughout the dilution series, and this was also the concentration utilized in the solvent-control wells. The plate was incubated at 37 °C for 4 h after 10 μL of 12 mM MTT stock solution (5 mg/mL in PBS, Invitrogen, Waltham, MA, USA; Cat.no. M6494) were added to each well. We drained the medium and added 100 μL DMSO (Sigma Aldrich, St. Louis, MO, USA; Cat. no.673439) for 20 min. The proportion of absorbance (at 570 nm) over the control value was used to calculate cell viability%. Three separate experiments carried out in triplicate were applied. 

### 4.5. Experimental Approach

Experiment 1 (stemness: proliferation, apoptosis, cell cycle, and migration): Rat BM-MSCs were divided into 3 groups as follows: vehicle (DMSO)-treated control cells (DMSO group), cells treated with 50 µg/mL WKO extract (WKO group), and cells treated with 50 µg/mL WKD extract (WKD group), and then the cells were re-incubated for 24 h. Doses of WKO and WKD were chosen based on the results of the MTT assay. 

Experiment 2 (premature senescence and ROS): Rat BM-MSCs were allocated into the following 4 groups: DMSO group, cells treated with 100 µM H_2_O_2_ (for induction of premature senescence) or 25 µM H_2_O_2_ (for induction of ROS) for 2 h to induce premature senescence (H_2_O_2_ group) [5], and cells treated with 50 µg/mL of either WKO (WKO group) or WKD (WKD group) extract 24 h before the addition of H_2_O_2_ for 2 h. After two PBS washes, all traces of H_2_O_2_ were removed, and the cells were re-cultured in new complete media for 24 h.

Experiment 3 (inflammation): Rat BM-MSCs were allocated into the following 4 groups: DMSO group, cells treated with 100 ng/mL LPS (for induction of inflammation) for 12 h (LPS group) [54], and cells treated with 50 µg/mL of either WKO (WKO group) or WKD (WKD group) extract 24 h before the addition of LPS for 12 h followed by media change, and then the cells were re-cultured in new complete media for 24 h.

### 4.6. Detection of Gene Expression by qPCR

We used a Trizol reagent (Invitrogen, Waltham, MA, USA, Cat# 15596026) to extract total RNA from BM-MSCs. RevertAid H Minus Reverse (Thermo Scientific, #EP04 51) was utilized to obtain cDNA from RNA. A Piko qPCR thermal cycler (Thermo Scientific, Waltham, MA, USA) and the integrated software were used to perform the qPCR assay and analyze the data. A 20 μL mix containing 2 μL cDNA, 2 μL forward and reverse primers (Table 3), 9 μL RNase water, and 10 μL Syber Green (Thermo Scientific) was prepared. We followed the thermal conditions per the manufacturer’s instructions and as previously described [55]. The gene expression (mean fold change) was normalized with the internal control *GAPDH* and measured by the 2^−ΔΔCt^ method compared to the control (DMSO) group.

### 4.7. Cell Cycle by Flow Cytometry

After trypsinization, rat BM-MSCs were fixed in 70% ethanol at −20 °C for 24 h and were incubated in an ice-cold buffer solution containing 100 µL propidium iodide (640932, Biolegend, San Diego, CA, USA) for 1 h in the dark. A flow cytometer (FACScan, BD Biosciences, One Becton Drive Franklin Lakes, NJ, USA) was utilized to check the cell cycle. The information was analyzed using FlowJo V.10 (BD Biosciences).

### 4.8. Intracellular Reactive Oxygen Species

By tracking the formation of the fluorescent dichlorofluorescein (DCF) from the non-fluorescent probe 2,7-dichlorofluorescein diacetate (DCFDA), we fluorometrically measured the levels of intracellular ROS as previously described [56]. In brief, BM-MSCs at 70% confluence were subjected to treatment with each extract for 24 h before exposure to 25 µM H_2_O_2_ for 2 h. The latter was used as an inducer for oxidative stress. The cells were washed with PBS, DCFDA (5 μM) was added, and they were incubated for 45 min at 37 °C in the dark. We used a fluorescent microplate reader to measure the fluorescence intensity of DCFDA (485 nm excitation and 535 nm emission) and the data were presented as intracellular ROS levels % of the control.

### 4.9. Determination of Antioxidant Enzyme Activity

The antioxidant activity of superoxide dismutase activity (SOD) and catalase (CAT) in BM-MSCs was calorimetrically evaluated by kits purchased from Biodiagnostics, Cairo, Egypt. BM-MSCs at 70% confluence were first treated with each extract for 24 h before the addition of 25 µM H_2_O_2_ for 2 h to induce ROS. At a pH of 10.2, SOD prevents the auto-oxidation of epinephrine into adrenochrome, allowing its concentration to be determined. The rate of H_2_O_2_ degradation at 240 nm is used as an indicator for CAT activity. As was previously documented [57], GPx activity was measured in BM-MSCs.

### 4.10. ELISA

The protein content of RIPA-lysed BM-MSCs was determined using a BCA kit and bovine serum albumin as a standard (Thermo Scientific). For caspase 3, we measured the cleavage of the caspase 3 substrate at 405 nm after incubating the purified protein (20–30 g) with a caspase 3 fluorogenic substrate (Alexis Biochemicals, Farmingdale, NY, USA). SIRT1 (MyBioSource, San Diego, CA, USA, Cat # MBS2600246; OD = 450 nm; CV of intra-assay precision = 8%; CV of inter-assay precision = 12%) and AMPK (MyBioSource, Cat # MBS2501430; OD = 450 nm; CV of intra-assay precision = 5%; CV of inter-assay precision = 5.5%) were also measured in lysed BM-MSCs. The results of the previously mentioned parameters were displayed as % of the control. However, the levels of pro-inflammatory cytokines IL1β (MyBioSource, Cat # MBS825017; OD = 450 nm; CV of intra-assay precision = 5.9%; CV of inter-assay precision = 5.0%), TNFα (MyBioSource, Cat # MBS2507393; OD = 450 nm; CV of intra-assay precision = 6.5%; CV of inter-assay precision = 5.5%), and NF-kB (MyBioSource, Cat # MBS453975; OD = 450 nm; CV of intra-assay precision = 10%; CV of inter-assay precision = 12%) were detected in conditioned media of BM-MSCs after induction of their release by LPS using specific rat ELISA kits as detailed in the manufacturer’s guidelines.

### 4.11. Statistical Analysis

Differences between groups were calculated using one-way analysis of variance (ANOVA) in GraphPad Prism 8 (GraphPad Software, Inc., LaJolla, CA, USA). The post hoc Tukey’s Honestly Significant Difference (HSD) test was used to compare means. The significance level used was *p* ≤ 0.05, and results were reported as mean ± standard error of the mean (SEM).

## 5. Conclusions

To the best of our knowledge, this is the first research to show that WKO and WKD extracts may increase stemness and decrease premature senescence of MSCs. WKO and WKD extracts increased MSC proliferation, migration, and antioxidant status and decreased MSC apoptosis, ROS, and inflammation. This beneficial effect could be due to WKO and WKD contents of flavonoids and phenolic compounds. As a result, these extracts may be employable as enhancers to improve the therapeutic effectiveness of MSC. However, further research is required to determine the efficacy and safety of WKO and WKD compounds in people, as well as to determine the specific mechanism of action of these compounds.

## Figures and Tables

**Table 1:** Flavonoids and phenolic compounds in WKO and WKD extracts.

Name	Retention Time (min)	Amount (µg/g)
**WKO extract**		
Gallic acid	3.489	21.81
3-Hydroxytyrosol	4.519	3.49
Ferulic acid	15.994	1.76
o-Coumaric acid	17.6	1.12
Resveratrol	19.21	9.41
Quercitin	20.9	1.70
Rosemarinic	22.1	1.43
Kaempferol	25.024	3.21
Total		43.93
**WKD extract**		
Quinol	3.15	52.26
Gallic acid	3.65	58.50
Catechol	5.464	21.93
p-Hydroxy benzoic acid	7.46	73.74
Catechin	9.069	11.70
Chlorogenic acid	9.47	38.47
Syringic acid	10.6	30.14
p-Coumaric acid	13.8	16.41
Benzoic acid	14.163	280.00
Ferulic acid	15.4	5.80
Rutin	16.27	19.02
Ellagic	16.8	6.50
o-Coumaric acid	17.3	3.90
Total		618.37

**Table 2:** Effect of WKO and WKD extracts on cell cycle of rat BM-MSCs.

Phase	Control (DMSO) Group	WKO Group	WKD Group
G0/G1	70.72± 2.50 ^b^	88.43± 0.9 ^a^	92.2± 1.12 ^a^
S	18.25± 2.05 ^a^	6.00 ± 0.07 ^b^	2.70 ± 0.05 ^b^
G2/M	8.50 ± 0.40 ^a^	0.15 ± 0.01 ^b^	0.02 ± 0.005 ^b^

Data were presented as mean ± SEM, *n* = 3. Values with different superscript letters (^a^ (highest value), ^b^ (lowest value)) in the same row are significantly different at *p* ≤ 0.05.

**Table 3:** Primers used for real-time PCR.

Gene	Forward Primer (5′–3′)	Reverse Primer (5′–3′)
Bax	ACACCTGAGCTGACCTTG	AGCCCATGATGGTTCTGATC
Caspase3	GGTATTGAGACAGACAGTGG	CATGGGATCTGTTTCTTTGC
Bcl2	ATCGCTCTGTGGATGACTGAGTAC	AGAGACAGCCAGGAGAAATCAAAC
MMP9	TCGAAGGCGACCTCAAGTG	TTCGGTGTAGCTTTGGATCCA
TIMP1	CGCAGCGAGGAGGTTTCTCAT	GGCAGTGATGTGCAAATTTCC
p53	ATGGCTTCCACCTGGGCTTC	TGACCCACAACTGCACAGGGC
p21	GAGGCCTCTTCCCCATCTTCT	AATTAAGACACACTGAATGAAGGCTAAG
SIRT1	GGCACCGATCCTCGAACAAT	CGCTTTGGTGGTTCTGAAAGG
AMPK	TGAAGCCAGAGAACGTGTTG	ATAATTTGGCGATCCACAGC
NrF2	CACATCCAGACAGACACCAGT	CTACAAATGGGAATGTCTCTGC
HO-1	GGAAAGCAGTCATGGTCAGTCA	CCCTTCCTGTGTCTTCCTTTGT
B-actin	AAGTCCCTCACCCTCCCAAAAG	AAGCAATGCTGTCACCTTCCC

## Data Availability

The data supporting the present findings are contained within the manuscript.

## Sample Availability

Samples of the compounds are not available from the authors.
